# Anti-EGFR antibody 528 binds to domain III of EGFR at a site shifted from the cetuximab epitope

**DOI:** 10.1038/s41598-021-84171-3

**Published:** 2021-03-11

**Authors:** Koki Makabe, Takeshi Yokoyama, Shiro Uehara, Tomomi Uchikubo-Kamo, Mikako Shirouzu, Kouki Kimura, Kouhei Tsumoto, Ryutaro Asano, Yoshikazu Tanaka, Izumi Kumagai

**Affiliations:** 1 Graduate School of Science and Engineering, Faculty of Engineering, Yamagata University, 4-3-16 Jonan, Yonezawa, 992-8510 Japan; 2 Graduate School of Life Sciences, Tohoku University, 2-1-1 Katahira, Aoba-ku, Sendai, 980-8577 Japan; 3 Laboratory for Protein Functional and Structural Biology, RIKEN Center for Biosystems Dynamics Research, Tsurumi-ku, Yokohama, 230-0045 Japan; 4 Department of Biotechnology and Life Science, Graduate School of Engineering, Tokyo University of Agriculture and Technology, 2-24-16 Nakacho, Koganei, Tokyo 184-8588 Japan; 5 School of Engineering, The University of Tokyo, 7-3-1, Hongo, Bunkyo-ku, Tokyo, 113-8656 Japan; 6 The Institute of Medical Science, The University of Tokyo, 4-6-1, Shirokanedai, Minato-ku, Tokyo, 108-8639 Japan

**Keywords:** Cancer therapy, Antibody therapy

## Abstract

Antibodies have been widely used for cancer therapy owing to their ability to distinguish cancer cells by recognizing cancer-specific antigens. Epidermal growth factor receptor (EGFR) is a promising target for the cancer therapeutics, against which several antibody clones have been developed and brought into therapeutic use. Another antibody clone, 528, is an antagonistic anti-EGFR antibody, which has been the focus of our antibody engineering studies to develop cancer drugs. In this study, we explored the interaction of 528 with the extracellular region of EGFR (sEGFR) via binding analyses and structural studies. Dot blotting experiments with heat treated sEGFR and surface plasmon resonance binding experiments revealed that 528 recognizes the tertiary structure of sEGFR and exhibits competitive binding to sEGFR with EGF and cetuximab. Single particle analysis of the sEGFR–528 Fab complex via electron microscopy clearly showed the binding of 528 to domain III of sEGFR, the domain to which EGF and cetuximab bind, explaining its antagonistic activity. Comparison between the two-dimensional class average and the cetuximab/sEGFR crystal structure revealed that 528 binds to a site that is shifted from, rather than identical to, the cetuximab epitope, and may exclude known drug-resistant EGFR mutations.

## Introduction

Epidermal growth factor receptor (EGFR) is a member of the closely related family of ErbB transmembrane protein tyrosine kinase receptors. Upon binding with its ligand, epidermal growth factor (EGF), EGFR triggers cellular growth and proliferation via phosphorylation signaling cascades^1,2^. In normal tissues, the function of EGFR is to ensure tissue homeostasis via a range of control mechanisms. EGF binds to the extracellular region of EGFR (sEGFR). Crystal structure analyses have revealed that sEGFR contains four domains and that EGF binds to the sEGFR sandwiched by domains I and III^3–6^. EGFR overexpression is widely observed in a variety of cancer cells, and in some cases, its signal is critical for cell survival and tumorigenesis. Therefore, the development of EGFR-targeting drugs to interrupt its signaling, either by blocking the EGF binding site or inhibiting its tyrosine kinase activity, is a promising approach for cancer therapy^7–10^. To date, three anti-EGFR therapeutic antibodies; namely cetuximab, necitumumab, and panitumumab, are available in the market^11^. Crystal structure analysis have revealed that these antibodies bind to domain III of EGFR and thereby inhibit binding of EGF, which in turn blocks the signaling and proliferation of cancer cells^12–14^.

Our studies have focused on an sEGFR binding clone 528^15,16^. To date, we have used antibody 528 as a basis to engineer various bispecific antibodies by combining the antigen-binding region of 528 and OKT-3, an anti-CD3 antibody^17–20^. These bispecific antibodies bridge EGFR on a cancer cell to CD3 on a T cell and induce effective T cell-mediated cancer cell killing. We have also reported the crystal structure of the antigen-free form of the 528 Fab fragment^21^. The structure revealed a concave surface to the antigen-binding site; however, detailed analysis of antigen binding is limited by the lack of structural determination of the complex between the 528 Fab fragment and sEGFR. Such detailed analysis of the recognition mechanism is crucial for developing highly effective therapeutic antibodies. In the present study, we performed structural analysis of the recognition of EGFR by 528 together with detailed binding analyses and confirmed the specificity of the binding to the tertiary structure by our demonstration that 528 cannot recognize heat-treated sEGFR samples.

## Results and discussions

### Dot blotting analyses using heat-treated sEGFR (the extracellular region of EGFR)

**Figure 1:**
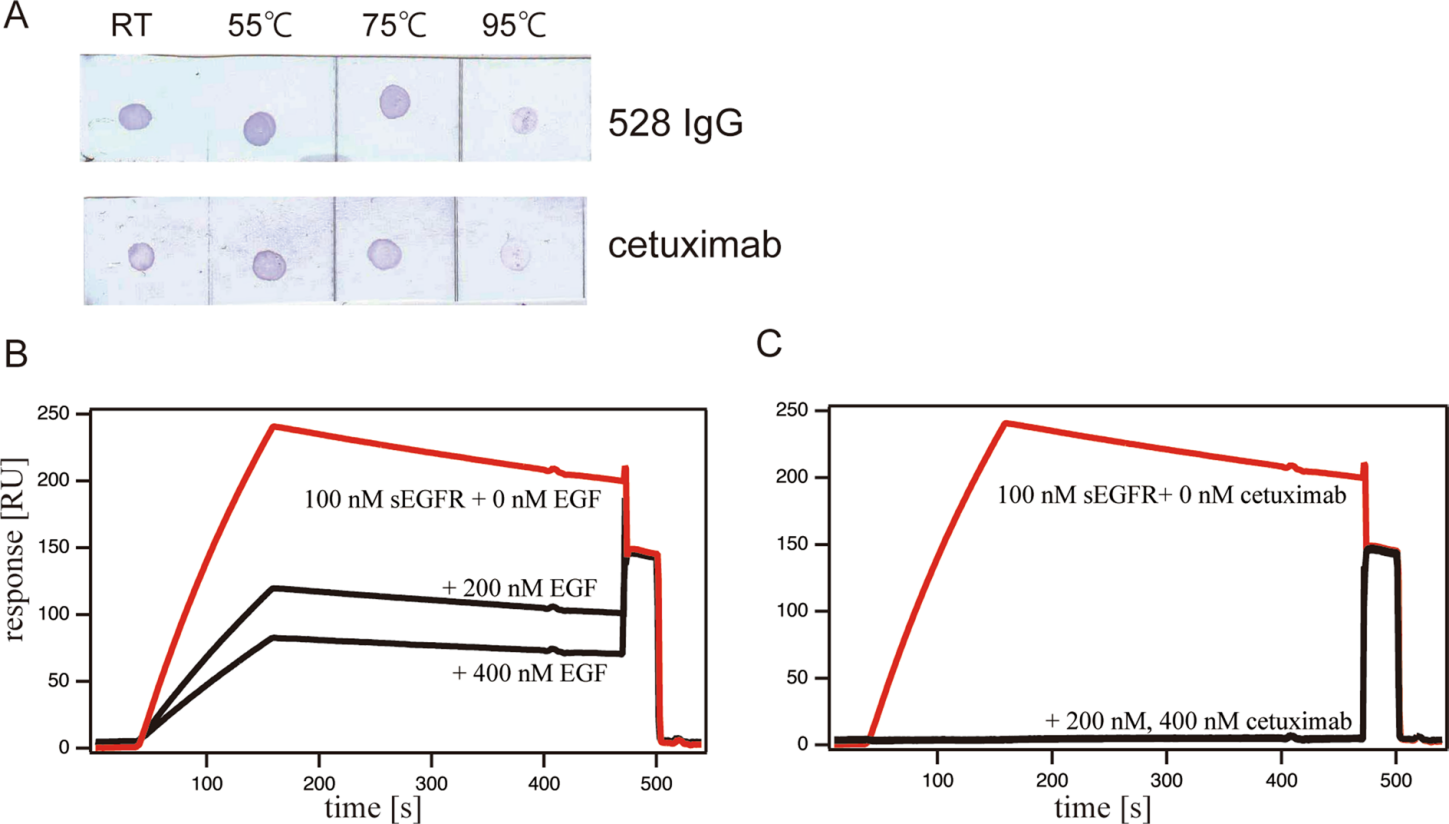
(**A**) Dot blot of heat-treated sEGFR detected by antibodies 528 and cetuximab. Each blotting image was a consecutive membrane strip without cropping. (**B**,**C**) Competitive Biacore assay between 528 and EGF (**B**) and between 528 and cetuximab (**C**) for binding sEGFR. sEGFR (100 nM) was loaded onto the antibody-immobilized sensor chips with or without its competitor.

Heat treatment of sEGFR will disrupt its tertiary structure. Therefore, these experiments allow the preliminary investigation of sEGFR recognition by the 528 antibody with respect to establishing whether the full tertiary structure is required for binding or whether 528 simply binds a short segment of an amino acid sequence. Dot blotting analyses were thus conducted using heat-treated sEGFR as antigen samples. Figure 1A shows that 528 can recognize dot-blotted sEGFR which has been heat-treated at 55 °C and 75 °C. On the contrary, for samples heated at 95 °C showed weaker staining, indicating decreased binding of 528 to sEGFR. This observation indicates that the integral sEGFR tertiary structure is required for recognizing the 528 antibody. Control experiments using cetuximab, which binds to the folded structure of domain III of sEGFR, as confirmed by crystal structure analysis, produced similar results, further confirming that the 528 antibody, similar to cetuximab, will recognize the tertiary structure of sEGFR. The results suggest that specific recognition of sEGFR by 528, to the extent that binding requires a folded soluble extracellular domain of the receptor. However, further evidence of specificity of binding is required.

### Competitive binding assay

Direct monitoring of the interaction between 528 and sEGFR was achieved using surface plasmon resonance (SPR). The experiments included the addition of competitor molecules for sEGFR to assess the specificity of binding by 528. Figure 1B,C show the results of a competitive binding experiment for 528 binding to sEGFR in the presence of EGF (the natural ligand) or cetuximab. As reported previously^15,22^, the addition of EGF or cetuximab to sEGFR before application to the sensor chips blocks the binding of 528 to sEGFR. These data suggest that 528 competes for binding to domain III of sEGFR and shares at least part of its binding site with that of EGF and cetuximab.

### 528 Fab binds to the surface of domain III in the vicinity of the cetuximab/EGF binding site

**Figure 2:**
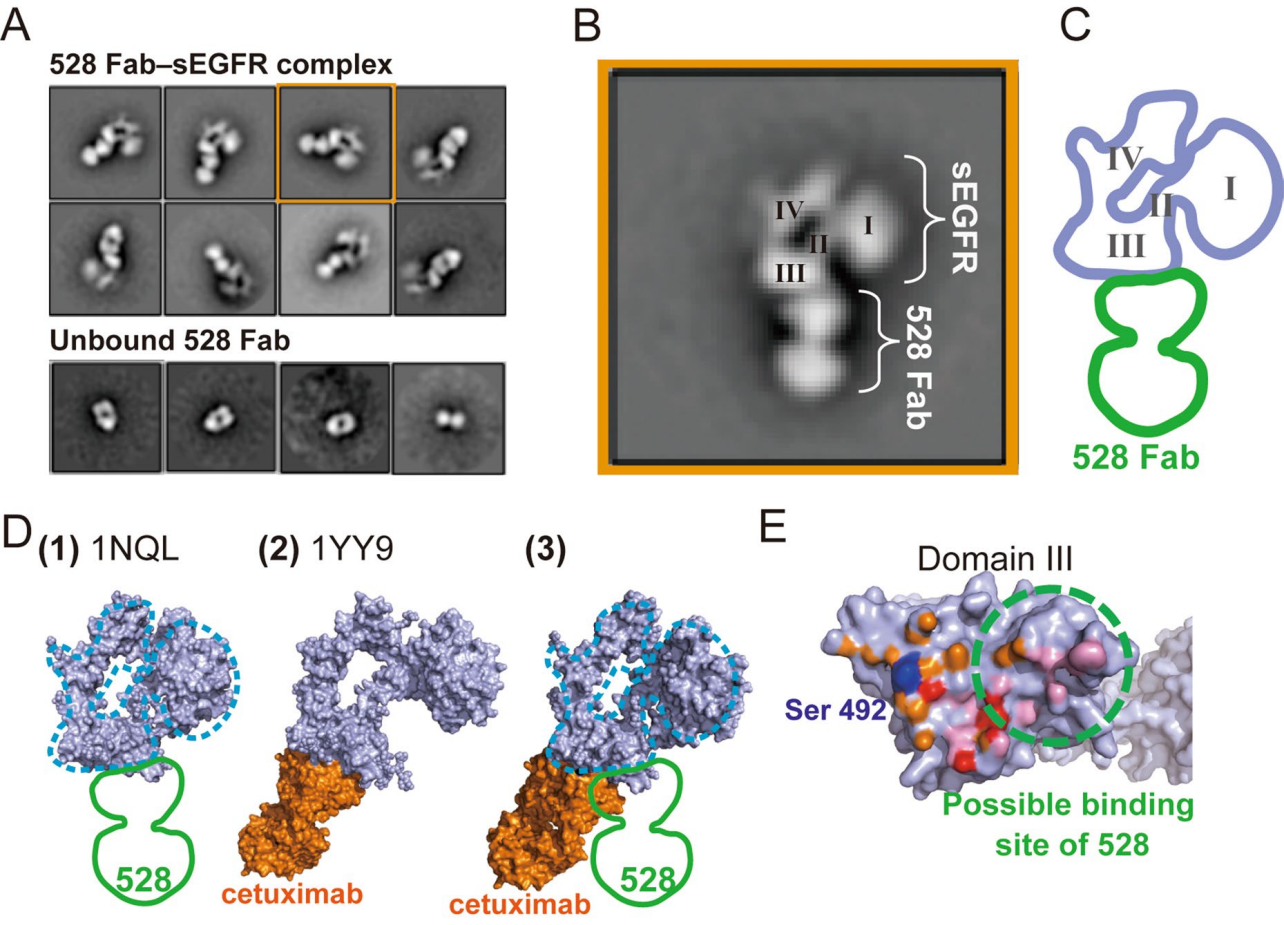
Negative-staining electron microscopy of the 528 Fab–sEGFR complex. (**A**) Two-dimensional (2D) class averages of the negatively stained 528 Fab–sEGFR complex. The purified complex was subjected to single particle image analysis via negative-staining electron microscopy. Upper panel: representative 2D class averages of the 528 Fab–sEGFR complex in their different orientation classes. Lower panel: dissociated 528 Fab. (**B**) Magnified representation of a 2D class average shown in (**A**) (orange square in (**A**), 90° rotated). Four domains of sEGFR are labeled. (**C**) An outline trace of the averaged image of (**B**) the sEGFR portion is shown in light purple and the 528 Fab portion is shown in green. (**D**) (1) A crystal structure of sEGFR (light purple, PDBID: 1NQL) overlapping with the outline trace (blue dashed line: sEGFR, green: 528 Fab). (2) A crystal structure of the cetuximab Fab–sEGFR complex (sEGFR: light purple, Cetuximab: orange, PDBID: 1YY9). (3) Cetuximab Fab onto the 1NQL sEGFR structure by superposing the domain III of both structures. The outline trace is overlapped onto the sEGFR structure (blue dashed line: sEGFR, green: 528 Fab). (**E**) Domain III surface from the view of the cetuximab side. Atoms within 3.5 Å from cetuximab and EGF are colored with orange (cetuximab), pink (EGF), and red (both). A possible binding area of 528 is indicated with a dashed green circle. Ser 492 is shown in blue.

**Figure 3:**
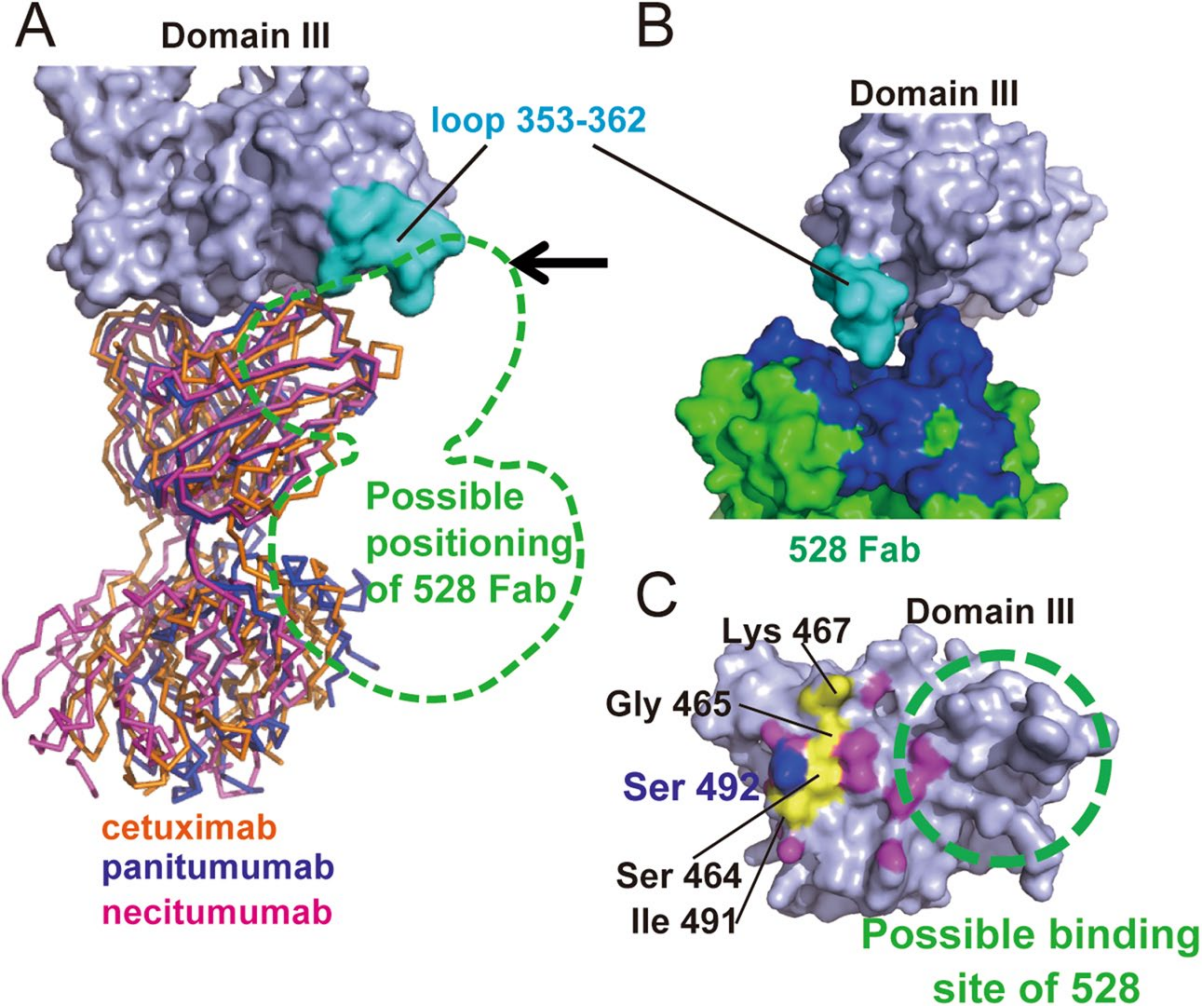
(**A**) Superposed anti-sEGFR antibody structure. Crystal structures of cetuximab (PDBID 1YY9, orange), panitumumab (PDBID 5SX4, blue), and necitumumab (PDBID 6B3S, magenta) antibodies complexed with domain III of sEGFR (light purple). Possible positioning of 528 Fab deduced from the electron micrograph images is indicated with a green dashed line. A black arrow indicates the viewpoint direction for (**B**). A loop comprising residues 353–362 is shown in cyan. (**B**) Manual docking model between domain III (light purple) and 528 (green). CDR loops are colored in blue and the 353–362 loop of sEGFR is in cyan. (**C**) Mutational residues of domain III induced by the cetuximab treatment (labeled with black characters). The possible binding site of 528 is indicated with a green dashed circle. Panitumumab epitope is colored with purple. The panitumumab epitope overlapping with the cetuximab-induced mutation is colored with yellow. Ser 492 is shown in blue.

To gain further detailed information on the recognition between sEGFR and 528, in the absence of successful crystallization, we pursued structural information using electron microscopy (EM) and single particle analysis. A sample of the 528 Fab–sEGFR complex on a carbon microscopy mesh grid was negatively stained and imaged using a Tecnai TF20 transmission electron microscope. The images obtained were subjected to single particle analysis. Upon two-dimensional (2D) classification, the particles were sorted into structurally homogeneous subgroups. Most particles converged into the representative 2D class averaged images of the 528 Fab–sEGFR complex, which clearly demonstrate the distinct mass of density corresponding to 528 Fab and sEGFR in a tethered state (Fig. 2A,B). The new structural information provided by these images clearly sheds light on the ability of the 528 antibody to inhibit growth factor signaling. For comparison, Fig. 2C shows the crystal structure of the sEGFR–cetuximab complex^12^. Figure 2D shows the surface of domain III of sEGFR, colored to indicate the intermolecular contact regions with cetuximab and EGF. Both proteins bind to the same surface of domain III and their contact sites are overlapped. Based on the EM images of the sEGFR-528 Fab complex (Fig. 2A–C) and the biochemical data demonstrating competition of 528 binding by EGF and cetuximab, it can be concluded that 528 binds to the same side of domain III of sEGFR as cetuximab and EGF. Interestingly, close inspection of the EM images and the sEGFR-cetuximab crystal structure revealed that, although both 528 and cetuximab bind to domain III, there is a marginal but distinct difference in their binding positions. This slight positional shift of the binding site of 528 is illustrated in Fig. 2D. The view from a certain direction of the crystal structure of free sEGFR (PDBID: 1NQL) is nicely overlapped with an outline trace of the class average shown in Fig. 2B (Fig. 2C,D(1)). On the other hand, an sEGFR crystal structure complexed with the cetuximab Fab fragment (Fig. 2D(2)) shows a little different orientation of domain III, which was not observed in the EM measurements. To compare the antibody binding sites between cetuximab and 528, the domain III structure of the complex structure (1YYE) was superposed onto that of the free sEGFR structure (1NQL). The resulting structure is shown in Fig. 2D(3) and has the sEGFR (light purple) of 1NQL with the cetuximab Fab portion (orange) from 1YY9. Overlapping the outline trace of Fig. 2C onto the sEGFR structure of Fig. 2D(3) clearly indicates that the binding site of 528 is shifted compared with that of cetuximab. Figure 2E shows the domain III surface view from the antibody side. The possible binding site of 528 overlaps with that of cetuximab and EGF. Such a difference in the binding sites is critical so as to offer 528 as a potential as a new antibody drug for the treatment of cancers that have become resistant to existing antibody therapies due to mutations in EGFR. For example, the three major anti-EGFR antibody drugs available in the market, namely cetuximab, necitumumab, and panitumumab, target relatively close epitopes (Fig. 3A)^12–14^. A cancer that has become drug-resistant due to a mutation within the shared epitope regions for these three antibodies, preventing their binding to EGFR, may still respond to 528 therapy. For example, the S492R mutation on domain III is a well-known mutation for cetuximab-treated patients who develop drug resistance^23,24^. Figure 2D shows that residue 492 of EGFR is located outside of the proposed binding epitope of 528 and it is, therefore, feasible that 528 may retain its binding activity to the S492R EGFR mutant and inhibit signaling and tumor growth. It should be noted that panitumumab also shows tolerance against the S492R mutation by accommodating the arginine sidechain to a central cavity at the antigen binding cleft (Fig. 3A,C)^14^. However, because of the high similarity of the epitopes between cetuximab and panitumumab, some cetuximab-resistant mutations, such as S464L, G465R, K467T, and I491M^24^, overlap with the panitumumab epitope (Fig. 3C). Thus, these mutations must affect the panitumumab binding to EGFR. On the other hand, the 528 epitope is shifted from the cetuximab epitope and cetuximab-induced mutation sites (Fig. 3C).

The previously determined crystal structure of a free form of 528 Fab revealed an interesting U-shaped cleft of approximately 15 Å, formed by the complementarity determining region (CDR) loops between heavy and light chains (Fig. 3B)^21^. This shape of the 525 Fab antigen-binding surface contrasts with that of cetuximab, which is relatively flat (Fig. 3A). Guided by our EM images of the sEGFR–528 Fab complex, manual inspection of the surface of domain III of sEGFR, in the region of the potential 528 binding site, identified a loop formed by residues 353–362, that forms a convex surface that may fit nicely within the cleft of 528 (Fig. 3B). Although this loop is a promising candidate for the epitope of 528, the determination of the structure of 528–sEGFR complex with atomic accuracy using x-ray crystallography or cryo-EM would provide the ultimate detail regarding the interaction. Such structural information would place our antibody engineering studies on firmer ground and expand their scope to wider design parameters and features, such as the development of bispecific and biparatopic antibodies.

## Methods

### Sample preparation

sEGFR with a C-terminal hexahistidine tag was expressed using the Chinese hamster ovary (CHO) cell expression system. The detailed expression method was as previously described^21^. The antibody samples were prepared from hybridoma cell cultures. The antibodies secreted into the culture medium were purified using protein A columns. Fab was prepared by using the Pierce Fab preparation Kit. EGF was purchased from Pepro Tech.

### Dot blotting analysis

Samples of sEGFR (1 μM in PBS) in microtubes were heat treated for 5 min at 55 °C, 75 °C, and 95 °C, respectively, and were subsequently dot-blotted onto a cellulose nitrate membrane. The membrane was blocked using 5% skimmed milk in PBS–Tween and was then incubated with 1 μg of primary antibody per 10 ml (528 IgG or cetuximab). A subsequent incubation with secondary antibody, anti-mouse IgG HRP conjugate for 528 or anti-human Fc IgG HRP conjugate for cetuximab, was followed by detection with an TMB chromogenic substrate (Kirkegaard & Perry Laboratories, Inc.). Between each step, the membrane was washed three times with PBS–Tween.

### Surface plasmon resonance (SPR) measurements

A competitive binding assay was performed using a Biacore T200 SPR instrument with CM5 sensor chips in HBS-EP buffer, pH 7.4. Antibodies were immobilized onto the sensor chips (2000 ~ 3000 RU) and 100 nM sEGFR samples with competitors were loaded for passing over the sensor chips. Binding sensorgrams were analyzed using the BIAevaluation program. Qualitative competition experiments were performed twice and all sensorgrams were reference subtracted.

### 528 Fab-sEGFR complex preparation, negative-staining electron microscopy and single particle analysis.

A 250 µL aliquot of 20 µM 528 Fab and a 300-µL aliquot of 20 µM sEGFR were mixed and incubated for 30 min at 4 °C. After filtration, a 500-µL aliquot was loaded onto a Superdex 200 10/300 GL column. The peak fractions, eluted at around 12 mL, were combined and used for further analyses (Supplemental Fig. 1A,B). A 3 µL portion of the purified 528 Fab–sEGFR complex was applied onto a continuous amorphous carbon surface attached to a R1.2/1.3 Cu 300 mesh grid (Quantifoil), which was glow-discharged at 5 mA for 30 s before use. A sample adsorbed to the carbon surface was negatively stained three times with 10 µL of 2% uranyl acetate solution. After the grid was completely dried, it was transferred onto a Tecnai TF20 transmission electron microscope (Thermo Fisher Scientific) operating at an accelerating voltage of 200 kV. The 528b Fab–sEGFR complex particles were imaged at a nominal magnification of × 80,000 and recorded using a Falcon II direct electron detector (Thermo Fisher Scientific) within the TF20. At this magnification, the corresponding objective pixel size at the specimen level is 1.3 Å. Automated data collection was performed using the Serial EM program^25^. Single particle image processing was performed using the RELION 3.0 program^26^. Briefly, 45,421 particles from 124 micrographs were picked using the AutoPick function in RELION. CTF parameters were estimated using the CTFFIND4 programyyy^27^. After 2D classification, images were converged to 2D class averages to obtain averaged 528 Fab–sEGFR complex images and isolated 528 Fab images (both in different orientations).

## Supplementary Information

Supplementary Information

## References

[1] Sigismund S, Avanzato D, Lanzetti L (2018). Emerging functions of the EGFR in cancer. Mol. Oncol..

[2] Yarden Y (2001). The EGFR family and its ligands in human cancer: Signalling mechanisms and therapeutic opportunities. Eur. J. Cancer.

[3] Ogiso H (2002). Crystal structure of the complex of human epidermal growth factor and receptor extracellular domains. Cell.

[4] Ferguson KM (2008). Structure-based view of epidermal growth factor receptor regulation. Annu. Rev. Biophys..

[5] Ferguson KM (2004). Active and inactive conformations of the epidermal growth factor receptor. Biochem. Soc. Trans..

[6] Ferguson KM (2003). EGF activates its receptor by removing interactions that autoinhibit ectodomain dimerization. Mol. Cell.

[7] Xu MJ, Johnson DE, Grandis JR (2017). EGFR-targeted therapies in the post-genomic era. Cancer Metastasis Rev..

[8] Wykosky J, Fenton T, Furnari F, Cavenee WK (2011). Therapeutic targeting of epidermal growth factor receptor in human cancer: Successes and limitations. Chin. J. Cancer.

[9] Jones S (2020). Targeting of EGFR by a combination of antibodies mediates unconventional EGFR trafficking and degradation. Sci. Rep..

[10] Martinelli E, De Palma R, Orditura M, De Vita F, Ciardiello F (2009). Anti-epidermal growth factor receptor monoclonal antibodies in cancer therapy. Clin. Exp. Immunol..

[11] Baldo, B. A. Monoclonal antibodies approved for cancer therapy. *Saf. Biol. Ther. Monoclon. Antibodies, Cytokines, Fusion Proteins, Horm. Enzym. Coagul. Proteins, Vaccines, Botulinum Toxins* 57–140 (2016)

[12] Li S (2005). Structural basis for inhibition of the epidermal growth factor receptor by cetuximab. Cancer Cell.

[13] Bagchi, A. *et al.* Molecular basis for necitumumab inhibition of EGFR variants associated with acquired cetuximab resistance. *Mol. Cancer Ther.* **17**, 521–531 (2018). 10.1158/1535-7163.MCT-17-0575 PMC5925748 29158469

[14] Sickmier EA (2016). The panitumumab EGFR complex reveals a binding mechanism that overcomes cetuximab induced resistance. PLoS ONE.

[15] Kawamoto, T. *et al.* Growth stimulation of A431 cells by epidermal growth factor: identification of high-affinity receptors for epidermal growth factor by an anti-receptor monoclonal antibody. *Proc. Natl. Acad. Sci.* **80**, 1337–1341 (1983). 10.1073/pnas.80.5.1337 PMC393592 6298788

[16] Gill GN (1984). Monoclonal anti-epidermal growth factor receptor antibodies which are inhibitors of epidermal growth factor binding and antagonists of epidermal growth factor binding and antagonists of epidermal growth factor-stimulated tyrosine protein kinase activity. J. Biol. Chem..

[17] Asano R (2006). Humanization of the bispecific epidermal growth factor receptor x CD3 diabody and its efficacy as a potential clinical reagent. Clin Cancer Res.

[18] Asano R (2013). Domain order of a bispecific diabody dramatically enhances its antitumor activity beyond structural format conversion: The case of the hEx3 diabody. Protein Eng. Des. Sel..

[19] Asano R (2014). Rearranging the domain order of a diabody-based IgG-like bispecific antibody enhances its antitumor activity and improves its degradation resistance and pharmacokinetics. MAbs.

[20] Asano R (2018). Comprehensive study of domain rearrangements of single-chain bispecific antibodies to determine the best combination of configurations and microbial host cells. MAbs.

[21] Makabe K (2008). Thermodynamic consequences of mutations in vernier zone residues of a humanized anti-human epidermal growth factor receptor murine antibody, 528. J Biol Chem.

[22] Sato JD (1983). Biological effects in vitro of monoclonal antibodies to human epidermal growth factor receptors. Mol. Biol. Med..

[23] Montagut C (2012). Identification of a mutation in the extracellular domain of the epidermal growth factor receptor conferring cetuximab resistance in colorectal cancer. Nat. Med..

[24] Arena S (2015). Emergence of multiple EGFR extracellular mutations during cetuximab treatment in colorectal cancer. Clin. Cancer Res..

[25] Mastronarde DN (2005). Automated electron microscope tomography using robust prediction of specimen movements. J. Struct. Biol..

[26] Zivanov J (2018). New tools for automated high-resolution cryo-EM structure determination in RELION-3. Elife.

[27] Rohou A, Grigorieff N (2015). CTFFIND4: Fast and accurate defocus estimation from electron micrographs. J. Struct. Biol..

