# Supplementary material for: Anti-EGFR antibody 528 binds to domain III of EGFR at a site shifted from the cetuximab epitope
Source: Sci Rep. 2021 Mar 11;11:5790. doi: 10.1038/s41598-021-84171-3 (PMC7952593; doi:10.1038/s41598-021-84171-3)
Supplement: Supplementary file 1 — Supplementary Information [file 41598_2021_84171_MOESM1_ESM.pdf]

Anti-EGFR antibody 528 binds to domain III of EGFR at a site shifted from the Cetuximab epitope

Koki Makabe[1], Takeshi Yokoyama[2,3], Shiro Uehara[2], Tomomi Uchikubo-kamo[3], Mikako Shirouzu[3], Kouki Kimura[4], Kohei Tsumoto[5, 6], Ryutaro Asano[4], Yoshikazu Tanaka[2], and Izumi Kumagai*[,4]

[1]Graduate School of Science and Engineering, Faculty of Engineering, Yamagata University, 4-3-16 Jonan, Yonezawa 992-8510, Japan

[2]Graduate School of Life Sciences, Tohoku University, 2-1-1 Katahira, Aoba-ku, Sendai 980-8577, Japan

[3]Laboratory for Protein Functional and Structural Biology, RIKEN Center for Biosystems Dynamics Research, Tsurumi-ku, Yokohama 230-0045, Japan

[4]Department of Biotechnology and Life Science, Graduate School of Engineering, Tokyo University of Agriculture and Technology, 2-24-16 Nakacho, Koganei, Tokyo 184-8588, Japan

[5]School of Engineering, The University of Tokyo, 7-3-1, Hongo, Bunkyo-ku, Tokyo 113-8656, Japan

[6]The Institute of Medical Science, The University of Tokyo, 4-6-1, Shirokanedai, Minato-ku, Tokyo 108-8639, Japan

*To whom correspondence should be addressed: kmiz@kuma.che.tohoku.ac.jp

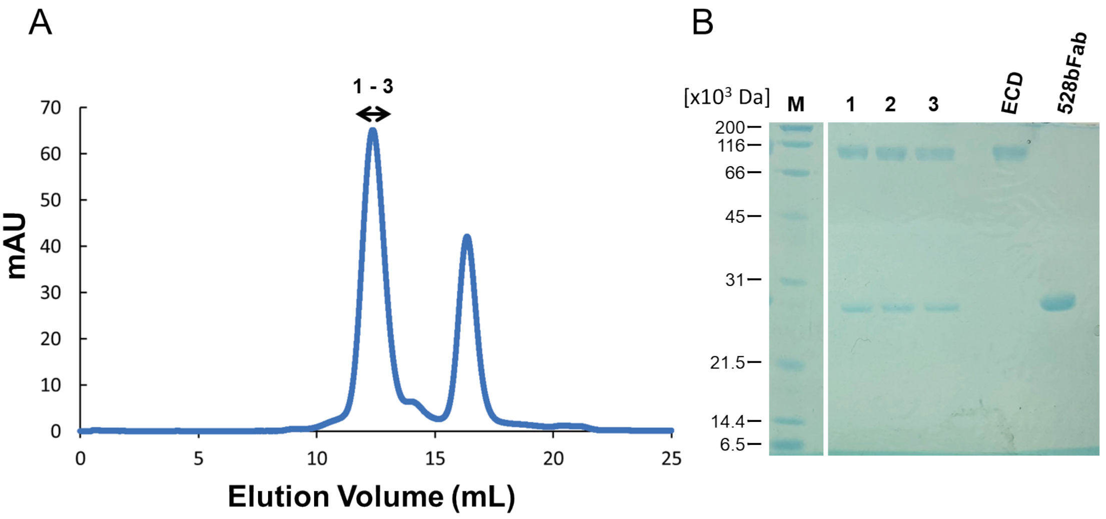

Supplementary Figure 1 Purification of 528b Fab-bound EGFR using gel filtration chromatography. (A) Chromatogram of SEC with Superdex 200 10/300 GL. (B) SDS-PAGE of the peak of SEC. As a reference, purified ECD and 528bFab were also loaded. A marker lane is shown for clarity.
